# Perch choice and substrate matching to the dorsal patterns of *Amphibolurus muricatus* lizards

**DOI:** 10.1093/beheco/araf129

**Published:** 2025-11-06

**Authors:** Jonathan W Salisbury, Richard A Peters

**Affiliations:** Animal Behaviour Group, La Trobe University, Melbourne, VIC 3086, Australia; Department of Ecological, Plant and Animal Sciences, School of Agriculture, Biomedicine & Environment, La Trobe University, Melbourne, VIC 3086, Australia; Animal Behaviour Group, La Trobe University, Melbourne, VIC 3086, Australia; Department of Ecological, Plant and Animal Sciences, School of Agriculture, Biomedicine & Environment, La Trobe University, Melbourne, VIC 3086, Australia

**Keywords:** camouflage, dorsal patterns, lizard, quantitative color pattern analysis

## Abstract

The backgrounds that cryptic animals choose will affect the efficacy of their camouflage. Most animals use a range of microhabitats consisting of a variety of substrates, vegetation and lighting conditions. As some of these will be better suited to facilitating camouflage than others, we expect cryptic animals to consider their conspicuousness when choosing a background to occupy. If the availability of backgrounds varies between populations of cryptic animals, then selective pressure on their coloration may also vary, resulting in intraspecific variation and presumably animals being better suited to the backgrounds locally available to them than those at other locations. In this study we investigate how backgrounds available to Jacky dragons (*Amphibolurus muricatus*) vary across their range, whether these lizards are occupying backgrounds that match well to their dorsal patterns, and how backgrounds compare to their dorsal patterns. Wild lizards were located and photographed along with the background they were found on, and other options available nearby. We compared lizards and backgrounds within their microhabitat as well as all backgrounds across all microhabitats. We found that lizards were not occupying the backgrounds that best matched their own patterns, that background options varied between locations, and that lizards from certain locations were occupying backgrounds closer matching to their own pattern than those from other locations. These outcomes provide interesting insight into the variance of local factors that influence the pattern phenotype, as well as how the relative need for camouflage might vary and be balanced with other needs.

## Teaser

Cryptic animals rely on camouflage, which depends on the backgrounds they choose. This study examined whether Jacky dragon lizards select backgrounds that match their body patterns using modified cameras and by modelling the visual capabilities of an avian predator. Results showed lizards did not always choose the best-matching backgrounds, and available backgrounds varied between locations. Findings reveal how local factors shape body patterns and how camouflage needs vary, balancing with other survival requirements in different habitats.

## Introduction

In most natural contexts, the efficacy of visual color and patterns employed by animals for camouflage and signaling is affected by the scene in which the animal is viewed (Stevens and Ruxton 2019). The microhabitats available to an animal in any location can vary in the types, arrangement, and distribution of visually relevant features, which include substrates, vegetation, structures, lighting, and weather conditions (Stevens and Merilaita 2011). For example, rainforest habitats occupied by both vertebrates and invertebrates often differ in fine-scale features across the landscape, shaped by variations in topography, soil composition, and water availability (Kinupp and Magnusson 2005). Habitats also vary within small areas as the vegetation, structure and lighting conditions differ significantly between the available microhabitats (ground level, mid-story, and upper canopy) (Valencia et al. 2004). Even ostensibly homogenous habitats, such as dry open forest, contain varied backgrounds for animals to occupy, including soil, rocks, leaf litter, fallen branches and vegetation (Bennett et al. 1991; de Pinho Werneck et al. 2009). Habitat features themselves often vary continuously in relevant traits such as coloration and functional patterning (Stevens and Merilaita 2009a) and the specific assembly of visually relevant components in a microhabitat, as well as how and where the individual is located within a scene, can affect how observers perceive color (Stevens and Merilaita 2009b; Dimitrova and Merilaita 2014).

Background matching is a form of camouflage in which an animal's coloration, pattern, or texture closely resembles the surrounding environment, making it harder for predators to detect it. For cryptic animals that employ background matching, increased habitat variability translates to discontinuous levels of conspicuousness to observers as they utilize the available habitat, which can result in increased predation threat (or decreased feeding success for predators) when occupying certain microhabitats (Michalis et al. 2017). This intrinsic relationship has been shown in many species utilizing coloration, such as bird nesting choice affecting clutch success (Troscianko et al. 2016; Stevens et al. 2017), and tree choice and orientation affecting predation risk in moths (Kang et al. 2015). To compensate for or to mitigate the risks associated with background choice, some animals can rapidly alter the color, pattern, or contrast of their body surfaces to match the background they occupy (Duarte et al. 2017), such as cuttlefish (*Sepia officinalis*, Mäthger et al. 2008) and gobies (*Gobius paganellus*, Smithers et al. 2018). However, most cryptic animals have fixed color patterns, or have a very limited capacity for short term change or are slow to change color (Green et al. 2019). Over generations camouflage phenotypes will presumably face selective pressure to better suit local conditions, and seasonal changes may be mitigated through ontogeny (Booth 1990), but most animals lack any significant short-term color plasticity to adapt to changes in the visual environment and varied backgrounds. Consequently, these species must maintain camouflage efficacy in other ways. Two divergent strategies are commonly observed in respect to this: specialize and match very well against a single or few background types, or compromise and adopt more generalized coloration that allows material if imperfect camouflage across a range of backgrounds available within the habitat (Merilaita et al. 1999; Houston et al. 2007; Hughes et al. 2019). Concurrently, individuals should maintain efficacy of their camouflage in the short term by selecting suitable backgrounds to occupy (Salisbury and Peters 2019). This behavioral maintenance of camouflage is often observed in cryptic animals, even by those that can rapidly change their coloration to suit (Barbosa et al. 2008; Smithers et al. 2018) and is critical to avoiding predation or detection for many camouflage strategies (Stevens and Ruxton 2019).

The association between phenotype and environment is conceptually simple, but mechanically complex. Superficially, the influence of observers (eg, predators) creates a selective pressure on camouflage, driving coloration toward better matching with the locally available backgrounds (Salisbury and Peters 2025a, 2025b). Under such assumptions, we should expect that different species utilizing the same background will converge in appearance. However, this is not the outcome in empirical studies (Stevens et al. 2014) as there are multiple factors involved and the selective forces of each interact in complex ways. For example, auxiliary functions of coloration, such as signaling and thermoregulation (Stuart-Fox and Moussalli 2009), and other needs of the individual (such as feeding and mating) may require compromises to camouflage efficacy due to background or locational requirements (or intentionally increase conspicuousness in the case of signaling and communicative functions; Roberts et al. 2007; Postema et al. 2023). As a result, we might predict that over time distinct populations within a species will vary phenotypically to better suit the specific combination of backgrounds, predators, and other contributing factors at their location (Camacho et al. 2020). This also implies that individuals from one population may be more conspicuous upon backgrounds available within the habitat of other populations or locations.

The relationship between the appearance of an organism and surrounding backgrounds is an important component of selection that has driven phenotypic diversity in cryptic animals (Camacho et al. 2020). Despite this, how cryptic animals relate to and choose backgrounds is underexplored, presumably due to the relative complexity of quantifying both camouflage and background traits. Compounding this, contemporary knowledge shows that camouflage strategies are often inherently linked with specific intended observers (Troscianko et al. 2017 ; Pike 2018). For example, avian predators are often shown to be perceptive into the ultra-violet (UV) spectrum (Lind et al. 2013) and therefore UV visual information should be considered when they are a potential source of selective pressure on a subject species. Thus, rigorous studies of camouflage should consider not just the animal itself, but the habitat in which it exists and from the perspective of relevant observers. Several studies have recently shown divergent selection of coloration to match local conditions whilst considering animal vision, including many lizard species (eg, McLean et al. 2014; Recknagel et al. 2023). Marshall et al. (2016) found that in a species of small lizard, dorsal patterns were better matched to local backgrounds than those at other locations, even for lizards that were geographically isolated in a relatively recent timescale. They also suggest that individuals living in more variable environments were worse matched in general to the available backgrounds, which may imply that a more generalist camouflage strategy can be beneficial in such contexts. Although great insight into pattern variation and background matching in lizards has been achieved, including accounting for visual system of intended receivers, a strong focus has been to quantify color and/or luminance (Marshall et al. 2016). Comparatively less work has sought to differentiate pattern (spatial arrangement of color/luminance differences) between individuals, and when undertaken, has not considered visual capabilities of ecologically relevant viewers (Raynal et al. 2022). What is lacking is an objective analysis of both color/luminance and pattern, as well as accounting for visual system differences and viewing distance.

**Fig. 1.**
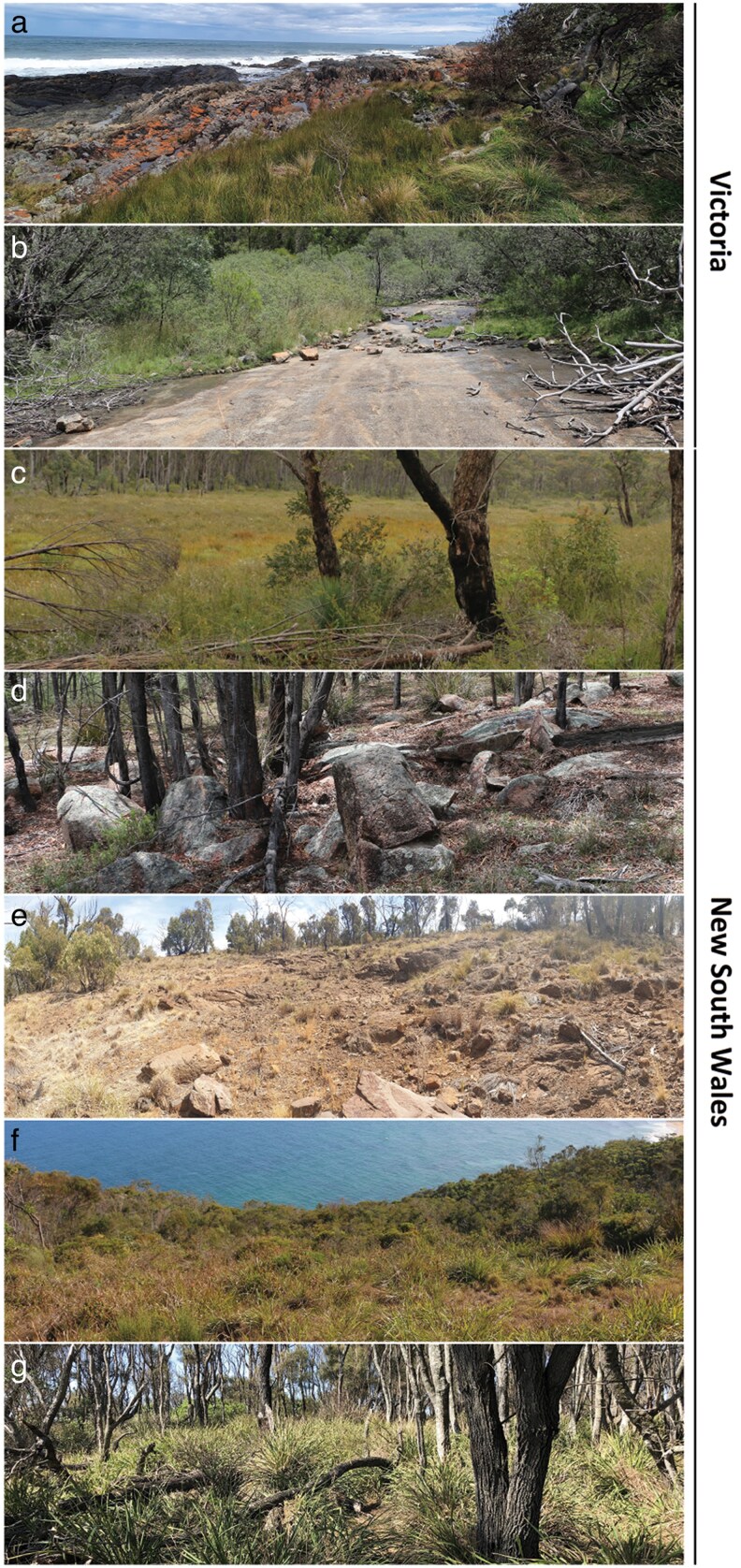
Variety in habitat and apparent visual complexity between Jacky dragon (*Amphibolurus muricatus*) population locations: (a) Croajingalong NP. (b) You Yangs RP. (c) Gibraltar Ranges NP. (d) Werrikimbe NP. (e) Warrumbungle NP. (f) Wyrrabalong NP. (g) Murramarang NP.

Jacky dragons (*Amphibolurus muricatus*) exhibit intraspecific variation of their dorsal patterns as a function of habitat type and location (Salisbury and Peters 2025a, 2025b) that might reflect some level of local adaptation to available backgrounds. Furthermore, if background options across these sampling locations also differ then it is reasonable to suggest that these lizards may match backgrounds available to them in their own microhabitat more so than those at other locations. Similar avenues of investigation have shown evidence of variation of coloration to suit local conditions in several species including crabs (Todd et al. 2006; Stevens et al. 2014), isopods (Merilaita 2001), and other squamates (Farallo and Forstner 2012), although none have explored these interactions with consideration to stakeholder predators. Jacky dragons are semi-arboreal, preferring to bask on raised objects, including tree limbs and rocks. We have previously found evidence that they will select for backgrounds that better match their dorsal patterns when given the choice between matching and non-matching backgrounds (Salisbury and Peters 2019), but they will utilize many backgrounds at times while hunting, defending territory, seeking mates, seeking shelter and moving throughout their territory (Peters and Evans 2007; Fitzsimons and Thomas 2018). Jacky dragons occupy a range of habitat types along the eastern and south-eastern coast of Australia, including dry forest, coastal heathlands and rocky outcrops (Cogger 2018). These habitats vary in structure and complexity (Fig. 1) and offer a diverse array of potential backgrounds for these lizards to choose to occupy and against which they might be viewed. Predation threat, visual complexity and other relevant factors may also vary between these locations.

In this study we investigated the backgrounds Jacky dragons choose to occupy in their microhabitat (their perch), as well as other backgrounds available to them nearby. We sampled multiple microhabitats in each of seven locations across the range of the species capturing genetic diversity and different habitat types. Our aims were to:

1. compare lizard patterns with the background against which the lizard was found (their perch) as well as other suitable basking options (backgrounds) nearby
2. quantify diversity in background options within the microhabitat of each lizard
3. compare the patterns of all lizards against all background options across all microhabitats in all locations sampled

We hypothesized that individual lizards would occupy the most suitable background in the immediate area, which we operationally defined to be the background most “like” their own pattern. Second, we hypothesized that microhabitat diversity in background options will vary as a function of one or more variables (genetic clade, sex, body size and habitat type), though we did not have a priori predictions on which factors will be relevant. Finally, we hypothesized that the degree of matching to backgrounds will not be uniform across the species range. To address these aims and hypotheses, we located Jacky dragons in a variety of locations and habitats (Table S1; Fig. 1) and photographed the lizard, the background they were occupying when found, as well as five distinct alternate background options in the vicinity of the lizard. We then quantified both lizard pattern and background features using micaToolbox (Troscianko and Stevens 2015; van den Berg et al. 2020) software suite for ImageJ software, incorporating Quantitative Color Pattern Analysis (QCPA; van den Berg et al. 2020) modeling the visual capabilities of the laughing kookaburra (*Daecelo novaeguineae*) as a representative predator of Jacky dragons (Allen et al. 2009) that is found in all study locations.

## Methods

### Study species

We located 52 wild Jacky dragons (*Amphibolurus muricatus*) and photographed the dorsal patterns of the lizard, the background they were occupying when first seen (their perch) as well as alternative basking backgrounds near to the captured lizard. We visited seven locations across the range of the species in south-eastern Australia (Fig. 1) that covered different habitat types and four of the five genetic clades (Pepper et al. 2014). Pepper et al. (2014) analyzed multiple nuclear loci and found strong support for geographically structured diversity. They identified five genetic groups with allopatric distributions down the east coast of Australia, and we sample four of these in the present study (see Table S1). Clade A was sampled in northern New South Wales (NSW) to the west of the Great Dividing Range (Werrikimbe National Park; Gibraltar Ranges National Park), while clade B was sampled just to the south of clade A and east of the Great Dividing Range (Wyrrabalong National Park). We sampled clade C lizards from inland NSW (Warrumbungles National Park), as well as on the NSW south coast (Murramarang National Park) and coastal eastern Victoria (Croajingalong National Park). Finally, clade D was sampled to the west of Melbourne, Victoria (You Yangs Regional Park). Lizards were visually located throughout the day (0800 to 1800), captured using standard techniques and then photographed as described below. The perch (background) the lizard was occupying when first located was then photographed, after which the lizard was released at that location. Five additional distinct background options within 5 m of the original perch were chosen and photographed, which allowed capture of a diverse range of backgrounds available to the lizards. In summary, each lizard dorsal image is associated with 6 background images (one of which is its chosen perch), with multiple of these lizard/background sets collected in each location (microhabitats), and multiple locations within each clade and habitat type. Images of three backgrounds were excluded due to file corruption resulting in 309 background images for 52 lizards.

### Image acquisition

#### Camera equipment

Photographs were taken using a Canon 7D Digital SLR camera, which had been modified to be sensitive to the Ultravoilet (UV) light spectrum (Camera Clinic, Melbourne, Australia), fitted with a Nikkor EL 80 mm f/5.6 lens suitable for UV photography. To capture visible spectrum images, a filter blocking infrared (IR) and ultraviolet wavelengths was applied to the lens (Baader UV/IR cut filter 2″). In effect this limited image information captured to the 420 to 385 nm wavelength range. Ultraviolet spectrum images were obtained by use of a UV pass filter (Baader U-Filter Visible/IR cut filter), with UV transmitted in the 300 to 400 nm range. The camera was calibrated for both VIS and UV spectrums using a set of 64-piece “Inscribe” artist pastels, excluding those with UV-fluorescent properties. Reflectance measurements for each pastel were taken outdoors under consistent sunlight using a JAZ spectrophotometer (Ocean Optics Inc.) and a standard probe. Multispectral photographs of the pastel array in natural light were then used to create a calibration profile. By combining the reflectance data of the color standards with the photographic information, the micaToolbox package could estimate the specific response of the camera and lens combination to various wavelengths, ensuring accurate color interpretation. All photographs included a white standard (layered white PTFE tape with a reflectance of 75% across 300 to 700 nm).

#### Photography

Photographs were taken in lossless RAW format using the camera's native ISO 200 to ensure image quality. Aperture was set at f/8 for sharpness, with exposure managed by adjusting shutter speed. Multiple images were captured at different exposure levels (−2, −1, and 0 steps) to ensure the best-exposed image could be selected. A manual focusing helicoid (25 to 55 mm) was used. For visible spectrum photos, focusing was done with a UV/IR cut filter via the viewfinder or live view. In UV photography with a UV-pass filter, manual focusing was done using live view as the viewfinder was ineffective. No focus shift was observed between image types, allowing seamless transitions between visible and UV photography without refocusing.

To photograph lizard patterns, the camera was mounted on a fully extended 6-foot tripod (Manfrotto 190XB) and positioned 1,500 mm above a stage designed for photographing lizards. Lizards were photographed in full shade or under an 800 mm diameter reflector when natural shade was unavailable. Using a Nikkor EL 80 mm f/5.6 enlarger lens, the setup ensured the lizard filled a significant portion of the frame. The camera was triggered remotely, and after capturing visible spectrum images, the filter was swapped to capture UV images of the same scene. If the lizard moved, the process was restarted. A scale was included in each image for reference. Photographs of backgrounds were taken perpendicular to the surface at a distance equal to the fully extended legs of the tripod in both visible and UV-spectrum. The physical set up and decision to leave backgrounds unimpacted meant that branches above the ground would be closer to the camera than those at ground level. Each photo contained a gray standard (also included in photographs of lizards) and scale and captured under natural lighting conditions, to best simulate the relevant lighting conditions (eg, natural forest lighting where predators may be present). Where the background was not evenly shaded, a photography shade was used such that even shade was applied across the scene, and that lighting was suitably diffuse.

### Image analysis

**Fig. 2.**
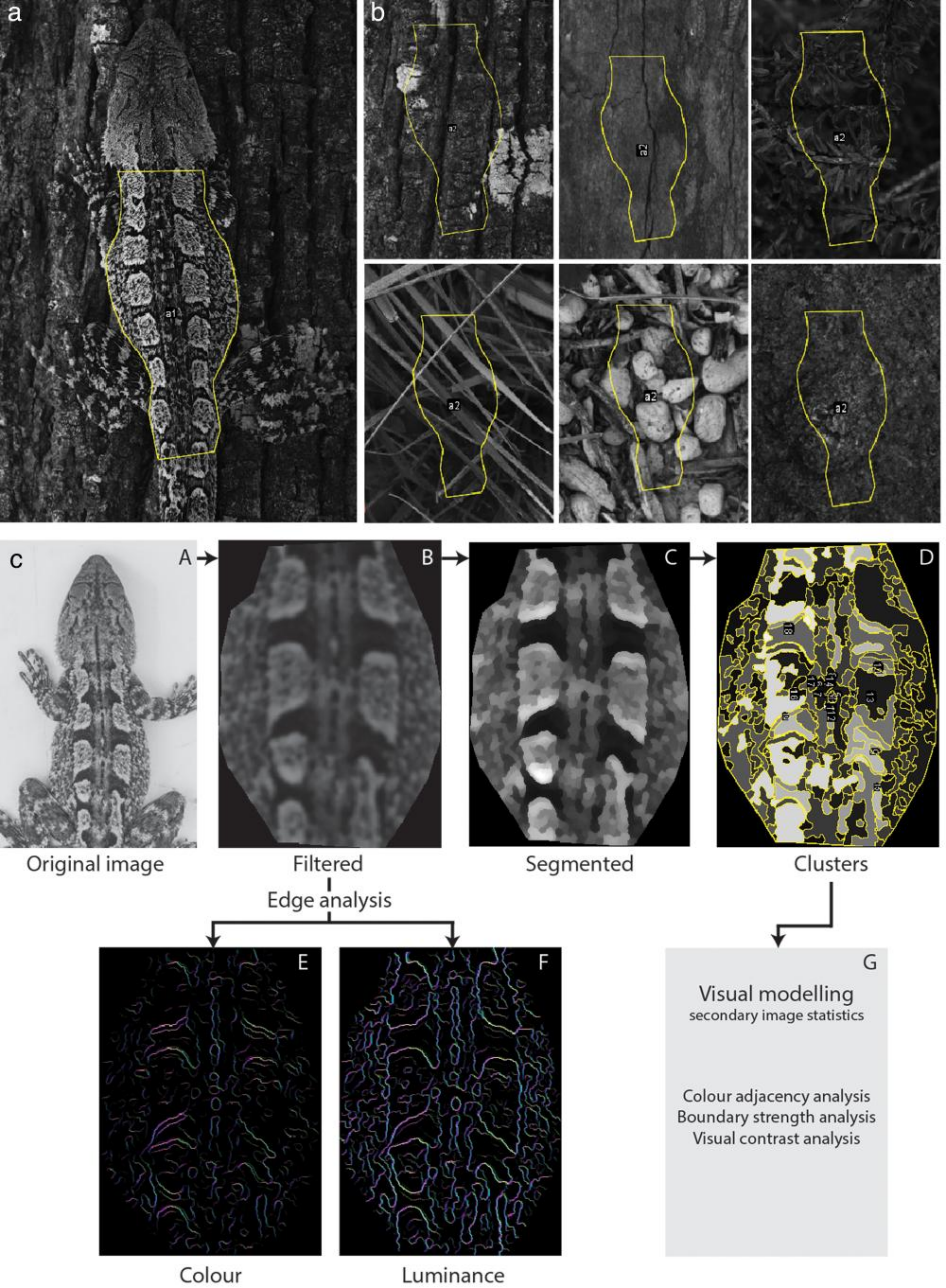
Image processing using the QPCA framework within the micaToolbox suite for ImageJ software. (a) Multispectral image created combining VIS and UV photographs and example ROI between the base of head and start of tail. (b) Scaled equivalent ROI applied to the background the lizard was utilizing when found (top-left) and other nearby background options. (c) QCPA workflow starts with the multispectral image (a) before it is filtered according to the acuity of the viewer and the viewing distance (b). Image segmentation based on RNL clustering (c) yields a set of distinct clusters for each image (D). Edge analysis uses the filtered version of the image quantifies the difference between neighboring regions of the image for both color (e) and luminance (f) channels. Secondary image statistics (g) are generated from the segmented image quantifying transitions between clusters.

We used QCPA and components of the micaToolbox (Troscianko and Stevens 2015; van den Berg 2020), a plugin module for ImageJ open-source software, to quantify lizard patterns and that of available backgrounds. This framework uses established color and pattern analysis procedures but also incorporates into the workflow correction for color vision capabilities, acuity and viewing distance. The visible-spectrum and UV images were combined to form multispectral images (32-bit) for analysis. To compare the dorsal patterns of each lizard to its respective background options, we restricted analysis to a region of interest (ROI), which was the area of dorsal pattern between the base of the head and the start of the tail (Fig. 2a). We then used the specific dorsal shape for each lizard to analyze an identical area of the perch it was found utilizing (Fig. 2b) and other background options (scaled appropriately for each background; Fig. 2c–g). The ROI overlaid on the background was oriented at random for large homogenous backgrounds (such as a rock) or in a manner consistent with how a lizard would be expected to be positioned on the given background (eg, lengthways for branches). Both the lizard dorsal ROI and each of the perch and background option ROIs were then assessed for pattern and coloration features using micaToolbox. We selected a single known predator of Jacky dragons, the laughing kookaburra (*Dacelo novaguineae*; Allen et al. 2009), and modeled their visual capabilities based on estimated cone types and their relative absorption profiles, as well as visual acuity values (cycles per degree, cpd). Laughing kookaburras possess violet sensitive (VS) visual systems and in the absence of specific cone sensitivities for the species we used generalized models for VS species (Long wave, λmax = 610 nm; Medium wave, λmax = 545 nm; Short wave, λmax = 475 nm; Violet sensitive, λmax = 415 nm; Double, λmax = 560 nm; Endler and Miekle 2005) and set acuity to 41 cycles per degree (cpd; Moroney and Pettigrew 1987). Gaussian acuity correction (non-square ROI's), negative replacement values of 0.001 and Weber fraction estimates of 0.05 (in absence of specific data) were used. We elected to use a single, biologically relevant viewing distance of 5 m.

After QCPA analysis of the dorsal and background ROI's, we extracted relevant pattern and color metrics for each image: number of clusters, counts within cluster, area of largest cluster, local edge intensity (color and luminance), as well as multiple color pattern measurements (Fig. 2c; Table S2). The color and discrimination thresholds of the kookaburra were used to segment the images resulting in clusters of the same color and luminance (panel D in Fig. 2c). The number of clusters, the area of the largest cluster and the number of patches of the same cluster type (counts within clusters) were used in our analysis. Local edge intensity analysis provides a measure of local edge contrast in four directions (horizontal, vertical and two diagonal directions) using the filtered (unsegmented) image (panel B in Fig. 2c), and from this we used the mean color and luminance values for the image whereby the maximum value from the four directions were used from each pixel in the image (panels E, F in Fig. 2c). Our final set of variables are secondary image statistics: color adjacency analysis (CAA), boundary strength analysis (BSA) and visual contrast analysis (VCA). A full description of the 45 secondary image statistics generated by QCPA is available elsewhere (van den Berg et al. 2020), and from this set, we chose a subset of twelve for further analysis. These are defined in Table S2 but provide information about color diversity, distribution and complexity (CAA variables), color and luminance pattern contrast (VCA) and color and luminance contrast at boundaries (BSA). However, since we had no prior expectations about which of these twelve variables deserved close attention, we applied principal components analysis (PCA) to simplify the dataset. For each observer, we extracted the color pattern variables and used the *prcomp* function to identify the principal components, centering and scaling the variables beforehand. To determine which components had statistical support, we employed the *PCAtest* function from the library of the same name (Camargo 2022) and selected the first three components (PC1-3) for further analysis.

### Data analysis

Data were examined in different ways as outlined below with all analyses undertaken in the R Statistical Environment (R Core Team 2020). Using the assessed pattern and color metrics, we first aimed to establish whether individual lizards were occupying the background option most like their own pattern (viewed from 5 m) before considering whether the level of matching between lizard and its basking background varied as a function of multiple factors. We then examined the extent of variation in background pattern within a given location before investigating how lizard pattern similarity to backgrounds varied at a species level by comparing patterns of all lizards with all backgrounds in all microhabitats at all locations. Output from the micaToolbox summarizing lizard and background patterns were grouped to define cluster, edge intensity, and visual model components of patterns. The cluster component comprised the number of clusters, the number of counts within clusters (summed across all clusters) and the area of the largest cluster. The edge intensity component comprised color and luminance intensity output, while the visual model component was defined by PC1-3.

#### Optimal background and available options

**Fig. 3.**
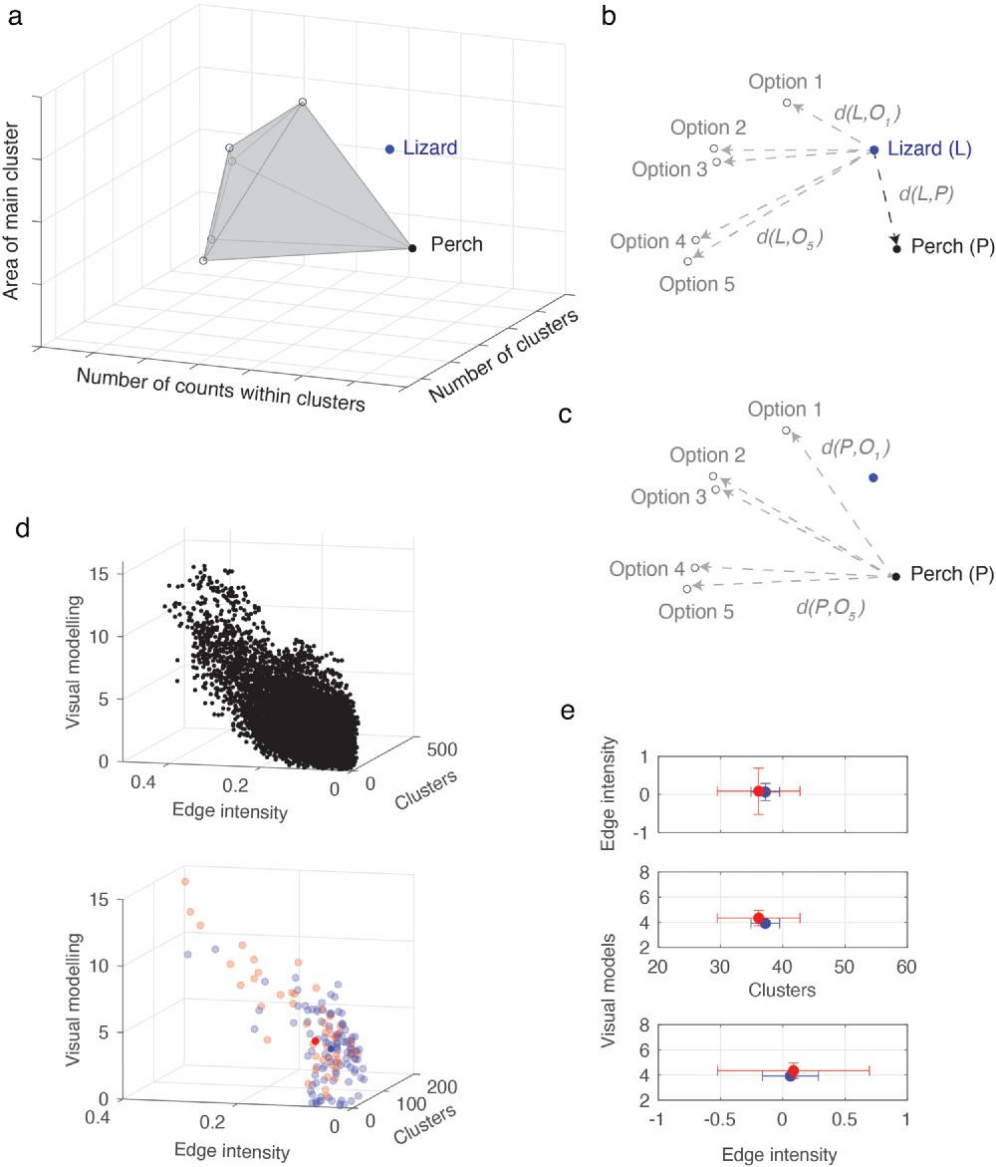
Analytical approach to examining lizard-background similarities. (a) Convex hull defined by the six background options for a given lizard is given by the gray shaded region. This example is for cluster data comprising number of clusters, number of counts within clusters and area of main cluster and the convex hull is computed in three-dimensions. (b) The Euclidean distance between lizard pattern detail and background options. The distance between the lizard (L) and background perch (P) on which lizard was found is represented as *d(L,P)*, with *d(L,O1)* and *d(L,O5)* representing the distance between the lizard and background options 1 and 5 respectively (distance between lizard and options 2 to 4 are not labeled). (c) distance between perch and each background option. (d) A total of 16,068 Euclidean distance values were computed for each lizard against all background options across all study locations for cluster, edge intensity and visual modeling data (top panel). A subset of distance values for one lizard in comparison to background options from two localities (bottom panel), with the mean distance for each locality shown in full color. The mean and standard error in two-dimensional space for cluster and edge intensity data (top panel), clusters and visual modeling data (middle panel) and edge intensity and visual modeling data (bottom panel).

We investigated whether the perch lizards were utilizing was the best available background option in their immediate surroundings when located. For the present analysis, we assumed that the optimal basking option for a given lizard, from a pattern matching perspective, would be the background closest in cluster (3D), visual model components (3D) or edge component (2D) space. Consequently, we computed the Euclidean distance between lizard pattern and basking options (Fig. 3a) and identified whether the background with the smallest distance was the background upon which the lizard was found. We used a proportion test separately for each component to determine whether the proportion of lizards within each clade found on the optimal background varied.

#### Basking background

We then looked for effects of snout-vent length (SVL), sex, clade or habitat type on how well lizards matched to backgrounds. The similarity between a lizard's pattern and the background upon which it was found (perch) was defined by the Euclidean distance between the two, and was undertaken separately for cluster, edge, and visual model components (Fig. 3b). We examined whether the distance varied as a function of sex (male, female), habitat (coastal heath, other), clade (A, B, C, D) and SVL. We fit generalized linear models using the *glm* function with interaction terms sex × habitat and sex × clade after log10 transforming the response variables where necessary (edge intensity and visual model data). The significance of models was first assessed with the *anova* function applying Type I sums of squares calculations to test interaction terms before the *Anova* function from the *car* package (Fox and Weisberg 2019) was used to test main effects implementing Type III sums of squares if at least one interaction term was significant (non-significant interaction terms removed) or Type II sums of squares when there were no significant interactions (interaction terms removed). We then selected the subset of lizards that were not on the optimal background and obtained the Euclidean distance between the basking background and the optimal background within the microhabitat from the set of distances computed between the perch and other background options (Fig. 3c). We repeated the statistical analysis above using this new response variable, with edge intensity and visual model data log10 transformed and cluster data square-root transformed.

#### Variation of available backgrounds

We examined how backgrounds themselves varied, both between and within microhabitats, locations, and habitat types. To examine variability in basking options available within a given microhabitat, we computed the three- or two-dimensional convex hull encapsulating all available backgrounds (Fig. 3a) separately for cluster, edge, and visual model components. We compared convex hull volume (cluster and visual model components) or area (edge intensity) as a function of sex (male, female), habitat (coastal heath, other), clade (A, B, C, D) and SVL. We fit a generalized linear model using the *glm* function with interaction terms sex × habitat and sex × clade after log10 transforming all three response variables. The significance of models was first assessed with the *anova* function applying Type I sums of squares calculations to test interaction terms before the *Anova* function from the *car* package was used to test main effects implementing Type III sums of squares if at least one interaction term was significant (non-significant interaction terms removed) or Type II sums of squares when there were no significant interactions (interaction terms removed).

#### Species wide matching to backgrounds

Lastly, we assessed how each lizard compared to each background photographed across all locations, and looked for any trends related to clade, habitat type or location. Consideration of lizard and background patterns to this point have been restricted to a given lizard within its own microhabitat. In the final treatment of the data, we computed the Euclidean distances between each of the 52 lizards and each of the 309 backgrounds photographed across all microhabitats of all locations, for a total of 16,068 lizard-background distance values each for cluster, edge, and visual model components (Fig. 3d). We first computed the mean (±SE) for each lizard in each location (Fig. 3d and e) and explored the data graphically in multiple ways: according to the clade to which the lizard belongs (4 clades), the habitat type (coastal heath, other) and the location of the background (7 locations) in which background was found. We selected the mean values computed above (N = 364) and compared distributions using Hotelling's T^2^ analysis for multivariate datasets in Matlab (Mathworks Inc). We examined the data as three-dimensional data as well as multiple two-dimensional data sets conducting multiple pairwise tests to compare withing clade, habitat type and location. Significance was determined after adjusting for multiple comparisons.

## Results

### Optimal background and available options

Three-quarters of lizards were found on tree branches or rock backgrounds (Figure S2a). A low proportion of lizards were found basking on the best matching background in the respective microhabitat (Table 1; Figure S2b). There was also no difference across clades in the likelihood that lizards would be found on the optimal background for each image analysis metric used (Table 1).

**Table 1.** The relative number of lizards from each clade that were occupying the optimal background in terms of cluster, edge intensity and visual modeling data.

Clade	N	Component		
		Cluster	Edge	Visual model
A	16	3	4	6
B	6	2	1	1
C	25	0	4	4
D	5	0	1	1
**Chi-square**		6.98	0.37	1.99
**Df**		3	3	3
***P*-value**		0.072	0.947	0.576

Results of proportion tests within clade for each component are also shown.

### Basking background

**Fig. 4.**
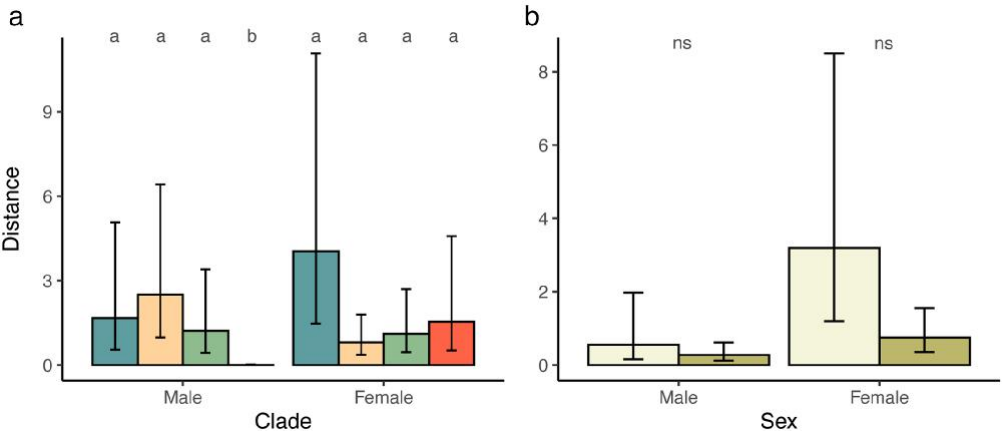
Estimated marginal means predicted from regression models for kookaburra observers at 5 m viewing distance reflecting the difference between chosen background and the best matching locally available background to the lizards' own pattern whereby higher values indicate larger differences between the chosen background and the best matching alternative. Results summarize the (a) interaction between sex and clade and for (b) sex and habitat for visual modeling data. Data for each sex in (a) is for clades a–d from left to right, while in (b) left bars depicts coastal heath, and right bars depicts other habitat types. Error bars are 95% CIs. Different letters above bars in (a) indicate pairwise differences.

We found no evidence that the level of matching between lizard and the basking background (perch) was affected by lizard size, sex, clade, or habitat type (Table 2). However, there were differences for the visual model component when the subset of lizards not on the optimal background was selected and comparisons made between the basking background and best available background (Table 3). The interactions between sex and habitat and sex and clade were both significant (Table 3; Fig. 4). Pairwise comparisons between clades within sex (Table S3) showed that males from clade D had significantly lower distance values than males from each of the other clades (Fig. 4a), meaning clade D males were a closer match to the background. All other comparisons for males, and all comparisons for females, were not significant. Pairwise comparisons between habitats within sex were not significant (Table S4; Fig. 4b).

**Table 2.** Statistical outcomes for analysis of degree of matching between Jacky dragon (Amphibolurus muricatus) dorsal patterns and the background upon which it was found.

Terms	SS type	Df	Test statistic^a^	Residual Df	Residual Dev.	*P*-value
*…*	*A. muricatus—Cluster traits*					
Sex	II	1	0.1321	…	…	0.1321
Habitat	II	1	0.9810	…	…	0.9810
Clade	II	3	0.0849	…	…	0.0849
SVL	II	1	0.3463	…	…	0.3463
Sex × Habitat	I	1	91.550	44	8109.7	0.4862
Sex × Clade	I	3	369.720	41	7740.0	0.5811
*…*	*A. muricatus—Edge intensity*					
Sex	II	1	0.6825	…	…	0.4087
Habitat	II	1	1.6172	…	…	0.2035
Clade	II	3	1.9005	…	…	0.5933
SVL	II	1	0.1140	…	…	0.7357
Sex × Habitat	I	1	0.0450	44	22.383	0.7726
Sex × Clade	I	3	0.2960	41	22.087	0.9079
*…*	*A. muricatus—Visual models*					
Sex	II	1	0.2632	…	…	0.2632
Habitat	II	1	0.9835	…	…	0.9835
Clade	II	3	0.5602	…	…	0.5602
SVL	II	1	0.2016	…	…	0.2016
Sex × Habitat	II	1	0.1655	44	16.155	0.4965
Sex × Clade	I	3	1.4794	41	14.675	0.2474

^a^Deviance for SS Type I and Chisquare for SS Type II or III.

**Table 3.** Statistical outcomes for analysis of difference between chosen background of Jacky dragon (Amphibolurus muricatus) and the best matching locally available background to the lizards' own pattern.

Terms	SS type	Df	Test statistic^a^	Residual Df	Residual Dev.	*P*-value
*A. muricatus—Cluster traits*						
Sex	II	1	3.066	…	…	0.080
Habitat	II	1	0.768	…	…	0.381
Clade	II	3	6.870	…	…	0.076
SVL	II	1	0.080	…	…	0.780
Sex × Habitat	I	1	1.445	39	78.679	0.405
Sex × Clade	I	2	1.415	37	77.264	0.713
*A. muricatus—Edge intensity*						
Sex	II	1	0.0129	…	…	0.9096
Habitat	II	1	1.6258	…	…	0.2023
Clade	II	3	5.1006	…	…	0.1646
SVL	II	1	0.3390	…	…	0.5604
Sex × Habitat	I	1	0.1676	34	26.319	0.6467
Sex × Clade	I	3	1.5858	31	24.733	0.5750
*A. muricatus—Visual models*						
Sex	**II**	**1**	**7**.**8368**	…	…	**0**.**00512**
Habitat	II	1	3.7868	…	…	0.05166
**Clade**	**II**	**3**	**37**.**5308**	…	…	**<0**.**0001**
SVL	II	1	0.3112	…	…	0.57692
**Sex × Habitat**	**II**	**1**	**2**.**6368**	**32**	**30**.**510**	**0**.**0102**
**Sex × Clade**	**II**	**3**	**18**.**936**	**29**	**11**.**574**	**<0**.**0001**

Lizards found occupying the “best” background not included in analyses. Bold denotes significant outcome.

^a^Deviance for SS Type I and Chisquare for SS Type II or III.

### Variation of available backgrounds

**Fig. 5.**
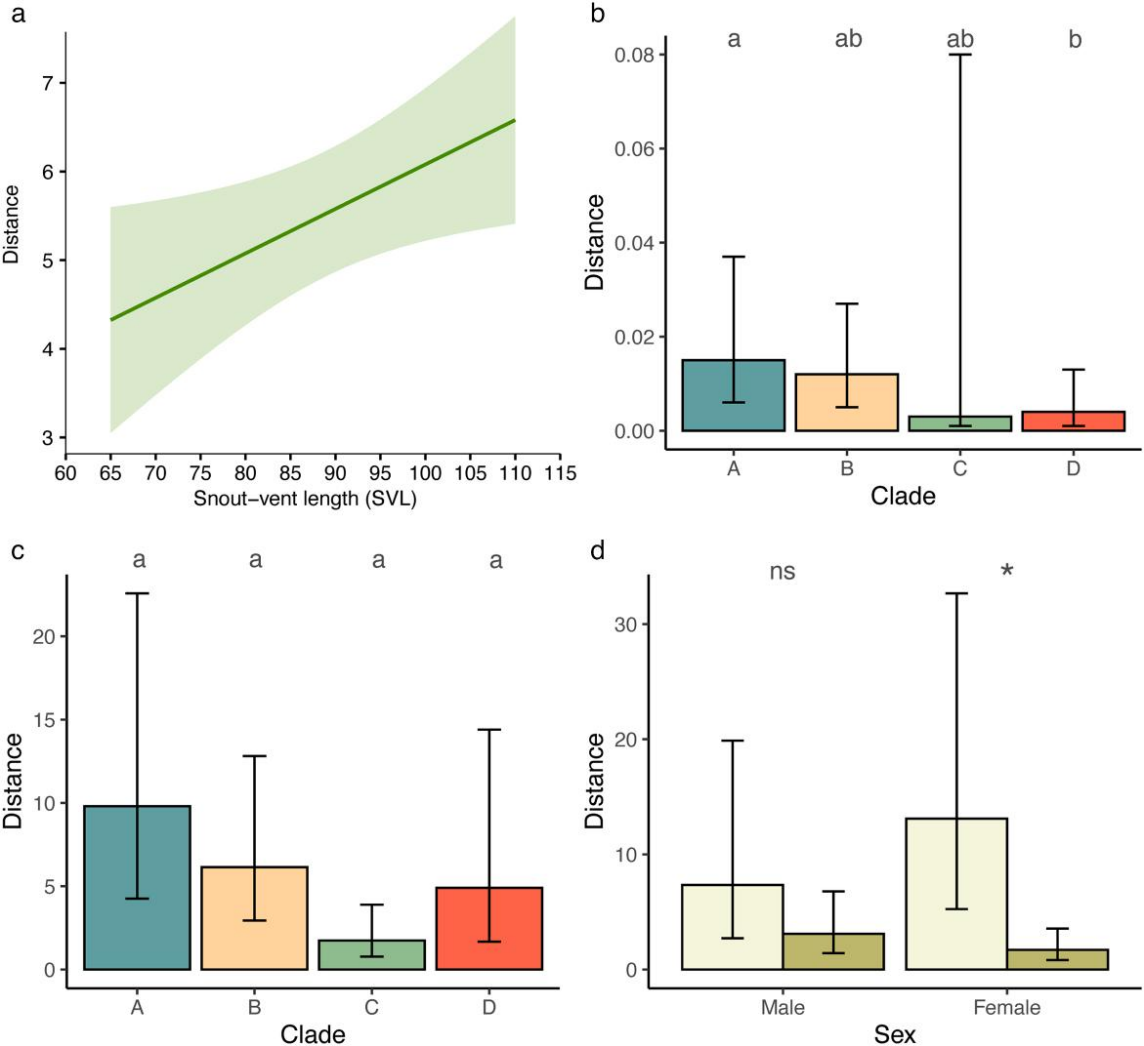
Estimated marginal means predicted from regression model for kookaburra observers at 5 m viewing distance reflecting the variability in background options with higher values indicating more variability. (a) Relationship between Euclidean distance and snout-vent length (SVL) for cluster data. The predicted value at each SVL is given by the solid line while the shaded region represents standard error of the predicted values. Predicted values for each clade for (b) edge intensity and (c) visual modeling data. (d) Predicted values for the interaction between sex and habitat for visual modeling data, where light green depicts coastal heath and dark green depicts other habitat types. Error bars in (b–d) are 95% CIs, with different letters or * above bars in indicate pairwise differences.

Background diversity did vary (Table 4), with evidence to support effects of lizard size, clade, and a sex × habitat interaction. Larger lizards were found occupying areas with more diversity in available backgrounds with respect to cluster traits (Fig. 5a). When assessing edge intensity, we found that background diversity varied across clades, specifically for clades A and D and a marginal effect for clades B and C (Fig. 5b; Table S5). We also observed an effect of clade (Fig. 5c) and an interaction effect for sex and habitat (Fig. 5d) when considering microhabitat level variability on the visual model component. None of the pairwise comparisons between clades reached significance after adjusting for the number of comparisons (Table S6), while backgrounds in coastal habitats displayed significantly more variation for microhabitats occupied by females; this contrast was not significant for males (Table S7).

**Table 4.** Statistical outcomes for analysis of available background variation and effects of Jacky dragon (Amphibolurus muricatus) factors.

Terms	SS type	Df	Test statistic^a^	Residual Df	Residual Dev.	*P*-value
*A. muricatus—Cluster traits*						
Sex	II	1	0.0002	…	…	0.9882
Habitat	II	1	0.1899	…	…	0.6630
Clade	II	3	7.2964	…	…	0.0630
**SVL**	**II**	**1**	**4**.**9273**	…	…	**0**.**0264**
Sex × Habitat	I	1	0.1971	44	99.415	0.7706
Sex × Clade	I	3	4.4078	41	95.007	0.5930
*A. muricatus—Edge intensity*						
Sex	II	1	0.0008	…	…	0.9774
Habitat	II	1	1.5019	…	…	0.2204
**Clade**	**II**	**3**	**15**.**6448**	…	…	**0**.**0013**
SVL	II	1	0.0097	…	…	0.9216
Sex × Habitat	I	1	0.9397	44	35.022	0.2856
Sex × Clade	I	3	1.2359	41	33.786	0.6823
*A. muricatus—Visual models*						
Sex	II	1	0.1063	…	…	0.7444
**Habitat**	**II**	**1**	**4**.**7684**	…	…	**0**.**0290**
**Clade**	**II**	**3**	**10**.**3862**	…	…	**0**.**0156**
SVL	II	1	1.0037	…	…	0.3164
**Sex × Habitat**	**II**	**1**	**4**.**1330**	**44**	**29**.**644**	**0**.**0150**
Sex × Clade	I	3	1.0561	41	28.588	0.6789

Bold denotes significant outcome.

^a^Deviance for SS Type I and Chisquare for SS Type II or III.

### Species wide matching to backgrounds

**Fig. 6.**
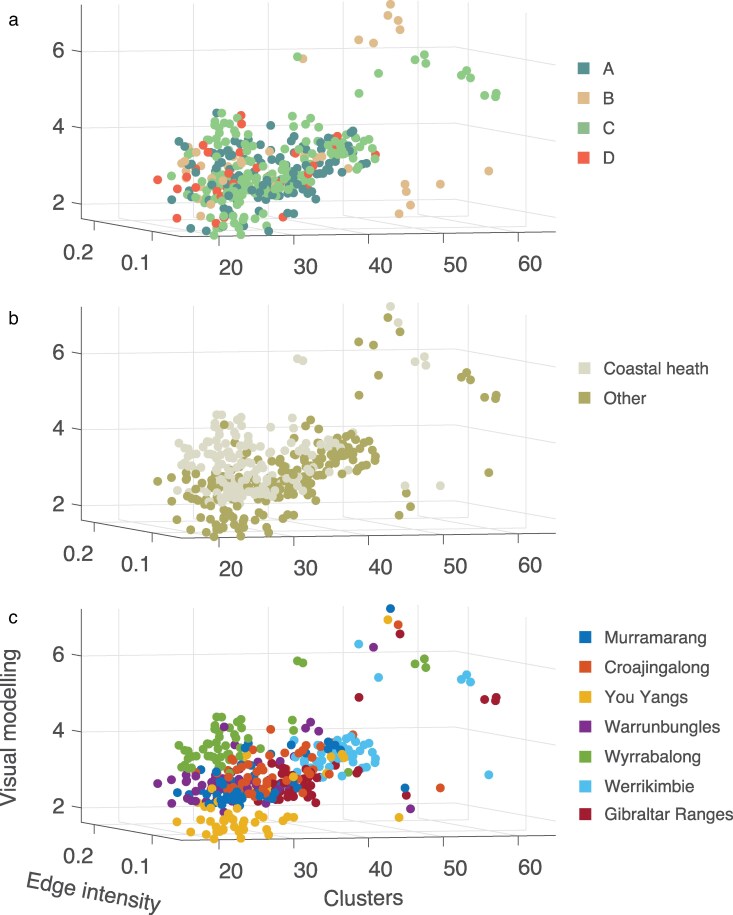
Three-dimensional plot of the Euclidean distance between each lizard and backgrounds from all locations in the study. Values are averaged within a given location for each lizard (variance not shown but see Fig. 4.7). The data are the same in each plot, but color coded according to (a) clade to which the lizard belongs, as well as the (b) habitat type and (c) location of the background.

**Fig. 7.**
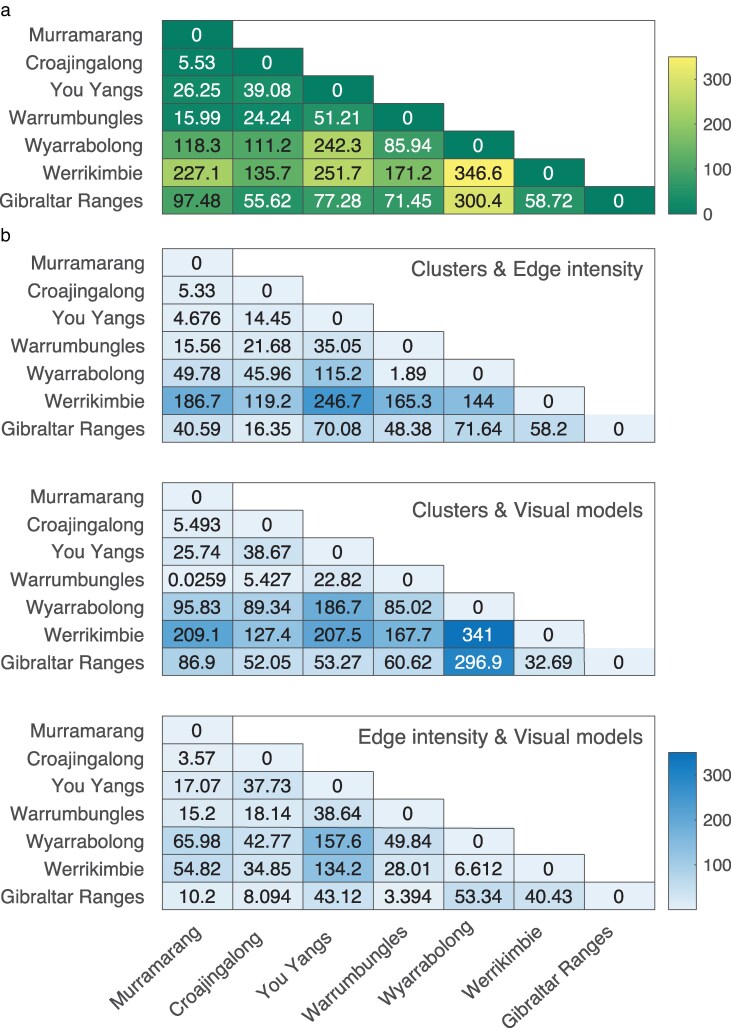
Heat maps depicting the chi-square statistic from Hotelling's T^2^ analysis of multivariate data comparing bivariate distributions between study locations. Data in (a) is from three-dimensional analysis while in (b) data is restricted to two-dimensions of clusters and edge intensity (top panel), clusters and visual models (middle panel) and (c) edge intensity and visual models. The degrees of freedom for each comparison within (a) and within (b) are the same (see Tables S8 and S9).

Values for each lizard and background were plotted in 2D space for edge intensity (color and luminance values) or in 3D space for clusters (number of clusters, counts within clusters and area of main cluster) and visual model components (PC1-3). Within each set the distance between lizard and all backgrounds was computed, before the mean (±1SE) for each lizard in each location was obtained (see Fig. 3). Mean lizard-background distances are presented in three-dimensions grouped by the clade to which the lizard belongs, the habitat type of the location and the location itself (Fig. 6a–c respectively; two-dimensional representations of the same data are shown Figure S3 along with standard error values). Clustering is apparent to some degree in each case but is clearest for the locality and habitat type of the background. We explored these distributions using Hotelling's T^2^ analyses to compare levels within each category in three- and two-dimensions (Tables S8 and S9 respectively). Considering the data in three-dimensions revealed that lizards from clades A, B and D were equivalent, while C differs from these. All but one of the pairwise contrasts for location was significant and the comparison between habitat types was also significant (Table S8). We examined the chi-square statistics more closely to provide insight into the relative differences between locations (Fig. 7a) and identified Werrikimbe and Wyrrabalong as standouts such that matching to backgrounds in these locations will be relatively less effective compared with each of the other locations (Werrikimbe is also the only location where distance measures between lizards and backgrounds are lower outside of the location compared to within for all three metrics; see Figure S4). To gain further insight into which pattern features contribute most to these differences, we repeated the Hotelling's T^2^ analysis in two-dimensional space (Table S9). The findings with respect to clade suggest that edge intensity differences might be responsible for clade C standing apart from the rest, while habitat differences are apparent across all three measures. The heat maps of chi-square statistics for the two-dimensional analyses presented in Fig. 7b implicate the same locations but also that cluster metrics are most influential.

## Discussion

The results of this study provide evidence for three general outcomes. First, contrary to our first hypothesis we found that Jacky dragons in general are not utilizing the “best” background available to them in the microhabitats they are occupying. Second, the backgrounds available to lizards across the various locations we investigated are not homogenous and differ both in their similarity to lizards and relative to other backgrounds available at the various locations. Finally, we provide evidence that the level of matching of backgrounds to lizard patterns is not the same between locations, with distinct groupings when considering backgrounds by habitat type and location, as well as when assessing lizards by clade.

Previous evidence has established that the degree of background matching significantly predicts predation attempts for species that rely on background-matching (Farallo and Forstner 2012). This suggests that Jacky dragons occupying backgrounds that closely resemble their own patterns would have advantages in evading predators, which is consistent with existing knowledge on camouflage function (Stevens and Merilaita 2011; Cuthill 2019). We describe elsewhere that Jacky dragons exhibit intraspecific variation of dorsal coloration and pattern traits (Salisbury and Peters 2025a, 2025b) and provide evidence in this study that backgrounds likewise vary. Salisbury and Peters (2019) showed that captive Jacky dragons would choose a patterned background over a more homogenous one, but the results of this study suggest that optimal background selection (in so far as we have measured) may not be the goal for this species. This contrasts with strategies of other lizards in which evidence for individual-specific concealment was strong (Marshall et al. 2016). There are a few explanations for why this difference might arise. First, we quantified pattern similarity rather than conspicuousness and it is entirely possible that the chosen background is as effective for concealing the lizards from avian predators as the optimal one. Second, in our earlier study (Salisbury and Peters 2019) proximity to cover had a higher effect size than background. Consequently, positioning of the background is likely to be an important mediator of background selection and possibly more important than background appearance per se. Of course, another central environmental feature are the predators themselves. Indeed, predation risk was a mediating factor for strength of concealment in Marshall et al (2016), with lizards at locations supporting fewer avian predators exhibiting comparatively reduced achromatic camouflage. This is suggested to be indicative of relaxed selection (Runemark et al. 2014). Our third explanation for Jacky dragons not using the optimal background is that they are less stringent in the choice of background because the threat of predation is not high.

The microhabitats inhabited by Jacky dragons were quite diverse. Consequently, precise matching to a particular background might not be the objective as lizards can be expected to utilize several distinct places to fulfill other functional tasks. Imperfect matching to a variety of potential backgrounds might indeed be the better strategy in such circumstances (Hughes, Liggins and Stevens 2019), particularly for organisms that are unable to change appearance over a short time frame. A so-called generalist strategy to background matching may offer potential benefits (Briolat et al. 2021), though the diversity of background options and predator behavior (Hughes et al. 2023), as well as background complexity itself (Rowe et al. 2021), are all important mediators of effectiveness. In our study, within microhabitat variability was not consistent across all individuals. For example, coastal heath locations tended to be more different to lizard appearance and featured greater variability in available options compared with other habitat types, although this appears to be more relevant to females than to males. This might render females more vulnerable in such environments that might constrain their behavior, limiting them to certain basking backgrounds. The potential differential impact on behavior for individuals in different habitats, or between sexes in the same habitat, is worthy of further consideration (Stevens and Ruxton 2019). If background matching is mediated by behavior, then our finding that larger lizards occupied microhabitats more different to their own appearance compared with smaller lizards might indicate microhabitat selection is driven by other functional behavior such as territorial defense and mating, with larger lizards being more willing to be seen. Indeed, kookaburras have been reported to exhibit sized based predation of another lizard species in favor of sub-adults (Blomberg and Shine 2000), and so large adult lizards may perceive lower risk. However, whether it is reduced vulnerability or prioritizing other functional behavior, or both, remains an open question.

Our approach enabled us to compare lizard patterns with potential basking backgrounds across the geographic range of the species. The level of matching between lizard appearance and backgrounds showed that habitat type was important, but importantly, the degree of matching is clustered based on location of the background. Backgrounds in the You Yangs showed a higher degree of matching to lizard patterns across all metrics (clusters, edge intensity and visual modeling) regardless of origin of the lizard; backgrounds from other locations showed lower levels of matching and greater within location variation. Locations differ in terms of plant species and background structure, so a functional difference in background matching seems plausible, with lizards in certain locations being less vulnerable than others. It is intriguing to consider the possibility that this reflects differences in relative predation risk, with higher risk in places where differences between lizard and backgrounds are lower (eg, You Yangs). This is an empirical question, but higher predation risk might result from the lower visual complexity of the open forest habitat, characterized by more open sightlines (Camp et al. 2012). Alternatively, increased predation threat results from greater predator diversity or density (Stevens and Merilaita 2009a). In either case, with fewer hiding spots and background options, individuals from the You Yangs may experience stronger overall selective pressure from visual predators (Iribarren and Kotler 2012; Camp et al. 2013), which could have led to the development of more closely matching patterns over time. This is further corroborated by our analysis of backgrounds compared to the “best” available background (excluding lizards already utilizing the best option). This analysis revealed an effect of sex and clade, specifically that males from clade D were occupying backgrounds significantly closer to the “best” option (according to visual model metrics) than males from other clades or females from any clade. Since the You Yangs falls within clade D, this provides further support for the hypothesis that individuals from this location face higher pressure to maintain effective camouflage compared to those at other locations (Marshall et al. 2016).

Background matching is only one of the potential functions of dorsal patterns of lizards. Camouflage efficacy is likely to be sacrificed to some extent when Jacky dragons are selecting backgrounds for other purposes (Kekäläinen et al. 2010; Garcia et al. 2013) that require specific placement within the habitat (eg, an exposed raised branch for thermoregulation, or to signal to conspecifics) to increase success in these capacities (and exert selective pressure). Any potential need for increased conspicuousness in terms of communication, for example, presumably also varies between locations, as increased vegetation density can directly translate to a more visually noisy environment and thus may make conspecific identification and interaction less effective (Ord et al. 2011). Previous studies of Jacky dragon signaling displays suggest these lizards intentionally increase their visibility to conspecifics, by positioning and orientating themselves prominently, and by increasing their display amplitude in visually noisy environments (Peters and Evans 2003; Barquero et al. 2015; Ramos and Peters 2017), providing evidence that communication is important, and that salience may be intentionally increased temporarily to this end. Matching of lizards to backgrounds in this study was worse in coastal heath habitat types, which is also regarded to be the more visually noisy habitat type for visual signaling (Ramos and Peters 2017), which maintains the possibility that camouflage efficacy is being sacrificed in such locations to compensate for more difficult conditions for visual signaling. An alternative consideration here is that lizards do not need to be closely matched to backgrounds in complex environments because of the impact that background complexity has on predator search behavior (Xiao and Cuthill 2016; Rowe et al. 2021). The three-dimensional environments lizards occupy might also influence predator search ability. Jacky dragons perch at varying heights above the ground and have other structures (branches, vegetation) between them and the predator that occlude them wholly or partially. Such microhabitats also generate dappled lighting and shadows that could affect detectability, as it does for dynamic visual signals (Bian et al. 2018). Further investigation into this habitat-predation-communication complex is needed to tease the selective pressures apart.

While there is a chance that these behaviors may also increase salience to predators, the more relevant insight here is that outcomes other than predation are potentially being considered when choosing a background to occupy and are influencing the phenotypes of camouflage. Incidentally, the dense vegetation that can cause a need for increased signaling ability may also offset any increased predation threats. Jacky dragons have been shown to respond rapidly to looming aerial stimuli (eg, swooping avian predators; Carlile et al. 2006), often by abandoning a perch and seeking refuge in vegetation, the proximity of which is a significant consideration for them when choosing backgrounds (Salisbury and Peters 2019). However, they respond less effectively to ambush predators (eg, snakes; Woo et al. 2017), and absconding would be less effective against this kind of threat. Nonetheless, vegetation offers refuge that breaks visual contact from predators (Potash et al. 2019) and physically shelters the individual (which can particularly effective against avian predators; Groner and Ayal 2001). Evidence shows that animals perceive and evaluate relative risk with respect to available vegetation (Embar et al. 2011; Potash et al. 2019) and lack of vegetation has been associated with changes in behavior in lizards to compensate (Pietrek et al. 2009). When considered within the context of the results of the present study, these observations imply that vegetation is an important supplement to camouflage for Jacky dragons, and habitats with more dense or readily available vegetation may alleviate avian predation risk to some extent.

Many factors can influence the phenotypes of animal color that we observe, and while predation is undoubtedly a common threat, the local conditions and traits of available backgrounds evidently have an effect (Stachowicz and Hay 2000). Overall, it is likely that how well animals match the available backgrounds is being mediated by a combination of predation threat and background availability/diversity, as well as possibly habitat complexity and vegetation availability. While previous studies have investigated background options and choices of other species (eg, Crisp et al. 1985; Heiling et al. 2005; Lovell et al. 2013; Smithers et al. 2018), they typically have not taken as broad and inclusive approach to the subject as we have done herein. Importantly, we did not measure conspicuousness but adopted a strategy to facilitate comparisons between lizard patterns and backgrounds not just in the same microhabitat but at locations across the distribution of the species. There is potential for this general methodology to explore variation between locations and quantify the detectability of out-of-place individuals, resulting in datasets to model relative visual predation threat in the context of relevant factors including genetic relatedness, habitat structure and predator-prey dynamics.

## Supplementary Material

araf129_Supplementary_Data

## Data Availability

Analyses reported in this article can be reproduced using the data provided by Salisbury and Peters (2025a, 2025b).
