# Supplementary material for: Perch choice and substrate matching to the dorsal patterns of Amphibolurus muricatus lizards
Source: Behav Ecol. 2025 Nov 6;36(6):araf129. doi: 10.1093/beheco/araf129 (PMC12636528; doi:10.1093/beheco/araf129)
Supplement: araf129_Supplementary_Data [file araf129_supplementary_data.pdf]

SUPPLEMENTRY MATERIAL

# Perch choice and background matching to the dorsal patterns of *Amphibolurus muricatus*

---

Supplementary material contained herein:

**Table S1:** Genetic clades (Pepper et al. 2014), habitat type and number of Jacky dragons

**Table S2** – Summary of notable output from QCPA analysis

**Table S3**– Pairwise contrasts for Perch-Option distance | Vis Mods : Clade within Sex

**Table S4** – Pairwise contrasts for Perch-Option distance | Vis Mods : Habitat within Sex

**Table S5** – Pairwise contrasts for habitat variation | Edge : Clade

**Table S6** – Pairwise contrasts for habitat variation | Vis Mod : Clade

**Table S7** – Pairwise contrasts for habitat variation | Vis Mods : Habitat within Sex

**Table S8** – Results of Hotelling's $T^2$ tests for comparing three-dimensional distributions

**Table S9** – Results of Hotelling's $T^2$ tests for comparing two-dimensional distributions

**Figure S1 –** Examples of lizard and background patterns

**Figure S2**– Background (perch) occupied by lizards and surrounding backgrounds

**Figure S3** - 2D plot of Euclidean distances between lizards and backgrounds from all locations

**Figure S4** – Lizard-background pattern differences by location …

**Table S1:** Genetic clades (Pepper et al. 2014), habitat type and number of Jacky dragons (*Amphibolurus muricatus*) captured, photographed, and released at each location in south-eastern Australia for analysis of dorsal patterns.

| State | Location | Clade* | Habitat type | No. lizards | No. perch options |
|---|---|---|---|---|---|
| Victoria | Croajingalong *N.P* | *C* | Coastal heathland | 7 | 42 |
| | You Yangs *R.P* | *D* | Dry open forest | 5 | 30 |
| New South Wales | Gibraltar Ranges *N.P* | *A* | Dry open forest | 9 | 54 |
| | Werrikimbe *N.P* | *A* | Dry open forest | 11 | 66 |
| | Warrumbungle *N.P* | *C* | Rocky outcroppings | 2 | 12 |
| | Wyrrabalong *N.P* | *B* | Coastal heathland | 6 | 36 |
| | Murramarang *N.P* | *C* | Coastal heathland | 20 | 120 |

***See Pepper et al. 2014**

**Table S2:** Summary of notable output from QCPA analysis of lizard dorsal patterns, after visual limitations of modelled observer are applied. See van den Berg et al. (2020) for full list and details of outputs.

| Output | Name | Description |
|---|---|---|
| Clusters | | Number of discreet patches of homogenous colour |
| | | Area of largest cluster |
| Counts | | Numbers of distinct patches within each cluster |
| Col.mean | Local edge contrast (colour) | Mean value across the image |
| Lum.mean | Local edge contrast (luminance) | Mean value across the image |
| CCA:Sc | Simpson colour diversity | Measure of how evenly colours are represented |
| CAA:Jc | Relative Simpson colour diversity | Measure of how evenly colours are represented independent of the number of colours |
| CAA:Hc | Shannon colour diversity | Alternate measure of colour diversity |
| CAA:Ht | Shannon transition diversity | Measure of regularity of transitions between different colours |
| CAA:C | Pattern complexity | Measure of pattern complexity based on transition frequencies |
| VCA:ML | Pattern luminance contrast | Weighted mean luminance of the image |
| VCA:MDmax | Pattern Dmax contrast | Weighted mean of Dmax contrast of the image weighted by the area of each colour |
| VCA:MSsat | Pattern RNL saturation contrast | Weighted mean RNL saturation of the image weighted by the area of each colour |
| BSA:BML | Luminance boundary strength | Weighted mean luminance difference of boundaries between colours |
| BSA:BMDmax | Dmax boundary strength | Weighted mean Dmax chromaticity difference of boundaries between colours |
| BSA:BMSsat | RNL saturation boundary strength | The Weighted mean RNL saturation difference of boundaries between colours |
| BSA:BMS | RNL chromaticity boundary strength | Weighted mean RNL colour difference of boundaries between colours |

**Table S3– pairwise contrasts for Perch-Option distance | Vis Mods : Clade within Sex**

| Contrast | estimate | SE | df | z.ratio | p.value |
|---|---|---|---|---|---|
| | | | Female | | |
| A - B | 1.608 | 0.711 | 29 | 2.262 | 0.174 |
| A - C | 1.288 | 0.856 | 29 | 1.504 | 0.606 |
| A - D | 0.963 | 0.479 | 29 | 2.009 | 0.283 |
| B - C | -0.321 | 0.486 | 29 | -0.659 | 0.987 |
| B - D | -0.645 | 0.730 | 29 | -0884 | 0.945 |
| C - D | -0.325 | 0.876 | 29 | -0.371 | 0.999 |
| | | | Male | | |
| A - B | -0.408 | 0.692 | 29 | -0.589 | 0.993 |
| A - C | 0.312 | 0.987 | 29 | 0.316 | 0.999 |
| **A - D** | **5.895** | **0.693** | **29** | **8.501** | **<0.001** |
| B - C | 0.720 | 0.698 | 29 | 1.032 | 0.893 |
| **B - D** | **6.302** | **0.896** | **29** | **7.037** | **<0.001** |
| **C - D** | **5.582** | **1.147** | **29** | **4.865** | **<0.001** |

**Table S4 – pairwise contrasts for Perch-Option distance | Vis Mods : Habitat within Sex**

| Contrast | estimate | SE | df | z.ratio | p.value |
|---|---|---|---|---|---|
| Female | 1.456 | 0.777 | 29 | 1.873 | 0.071 |
| Male | 0.708 | 0.911 | 29 | 0.778 | 0.443 |

**Table S5 – pairwise contrasts for habitat variation | Edge : Clade**

| Contrast | estimate | SE | df | z.ratio | p.value |
|---|---|---|---|---|---|
| A - B | 0.207 | 0.672 | 45 | 0.308 | 0.999 |
| A - C | 1.532 | 0.837 | 45 | 1.831 | 0.368 |
| A - D | 1.313 | 0.470 | 45 | 2.793 | 0.045 |
| B - C | 1.326 | 0.511 | 45 | 2.595 | 0.074 |
| B - D | 1.106 | 0.753 | 45 | 1.470 | 0.619 |
| C - D | -0.219 | 0.922 | 45 | -0.238 | 0.999 |

**Table S6 – pairwise contrasts for habitat variation | Vis Mod : Clade**

| Contrast | estimate | SE | df | z.ratio | p.value |
|---|---|---|---|---|---|
| A - B | 0.468 | 0.617 | 44 | 0.758 | 0.973 |
| A - C | 1.732 | 0.770 | 44 | 2.249 | 0.165 |
| A - D | 0.693 | 0.440 | 44 | 1.576 | 0.543 |
| B - C | 1.264 | 0.472 | 44 | 2.681 | 0.060 |
| B - D | 0.225 | 0.696 | 44 | 0.324 | 0.999 |
| C - D | -1.039 | 0.847 | 44 | -1.226 | 0.786 |

**Table S7 – pairwise contrasts for habitat variation | Vis Mods : Habitat within Sex**

| Contrast | estimate | SE | df | z.ratio | p.value |
|---|---|---|---|---|---|
| Female | 2.033 | 0.741 | 44 | 2.743 | 0.009 |
| Male | 0.861 | 0.771 | 44 | 1.1116 | 0.270 |

**Table S8:** Results of Hotelling's $T^2$ tests for comparing three-dimensional distributions of distances between lizard patterns and background options from all sites (see text for details).

| | | n1 | n2 | T2 | Chi-sqr. | df | P | Significant* |
|---|---|---|---|---|---|---|---|---|
| Clades | | | | | | | | |
| A | B | 112 | 42 | 10.15 | 10.15 | 3 | 0.01730 | No |
| A | C | 112 | 175 | 59.25 | 59.25 | 3 | 0.00000 | Yes |
| A | D | 112 | 35 | 5.15 | 5.15 | 3 | 0.16099 | No |
| B | C | 42 | 175 | 53.10 | 53.10 | 3 | 0.00000 | Yes |
| B | D | 42 | 35 | 10.91 | 3.54 | 3 | 0.01870 | No |
| C | D | 175 | 35 | 19.44 | 19.44 | 3 | 0.00022 | Yes |
| Locality | | | | | | | | |
| 1 | 2 | 52 | 52 | 5.53 | 5.53 | 3 | 0.13662 | No |
| 1 | 3 | 52 | 52 | 26.25 | 26.25 | 3 | 0.00001 | Yes |
| 1 | 4 | 52 | 52 | 15.99 | 15.99 | 3 | 0.00114 | Yes |
| 1 | 5 | 52 | 52 | 118.28 | 118.28 | 3 | 0.00000 | Yes |
| 1 | 6 | 52 | 52 | 227.14 | 227.14 | 3 | 0.00000 | Yes |
| 1 | 7 | 52 | 52 | 97.48 | 97.48 | 3 | 0.00000 | Yes |
| 2 | 3 | 52 | 52 | 39.08 | 39.08 | 3 | 0.00000 | Yes |
| 2 | 4 | 52 | 52 | 24.24 | 24.24 | 3 | 0.00002 | Yes |
| 2 | 5 | 52 | 52 | 111.16 | 111.16 | 3 | 0.00000 | Yes |
| 2 | 6 | 52 | 52 | 135.66 | 135.66 | 3 | 0.00000 | Yes |
| 2 | 7 | 52 | 52 | 55.62 | 55.62 | 3 | 0.00000 | Yes |
| 3 | 4 | 52 | 52 | 51.21 | 51.21 | 3 | 0.00000 | Yes |
| 3 | 5 | 52 | 52 | 242.33 | 242.33 | 3 | 0.00000 | Yes |
| 3 | 6 | 52 | 52 | 251.72 | 251.72 | 3 | 0.00000 | Yes |
| 3 | 7 | 52 | 52 | 77.28 | 77.28 | 3 | 0.00000 | Yes |
| 4 | 5 | 52 | 52 | 85.94 | 85.94 | 3 | 0.00000 | Yes |
| 4 | 6 | 52 | 52 | 171.23 | 171.23 | 3 | 0.00000 | Yes |
| 4 | 7 | 52 | 52 | 71.45 | 71.45 | 3 | 0.00000 | Yes |
| 5 | 6 | 52 | 52 | 346.59 | 346.59 | 3 | 0.00000 | Yes |
| 5 | 7 | 52 | 52 | 300.44 | 300.44 | 3 | 0.00000 | Yes |
| 6 | 7 | 52 | 52 | 58.72 | 58.72 | 3 | 0.00000 | Yes |
| Habitat | | | | | | | | |
| Coastal heath | Other | 156 | 208 | 143.36 | 143.36 | 3 | 0.00000 | Yes |

*** Significance after adjusting for repeated pairwise contrasts**

**Table S9 (next page):** Results of Hotelling's $T^2$ tests for comparing three-dimensional distributions of distances between lizard patterns and background options from all sites (see text for details).

| Hotelling's T2 using 2 variables | | | | | | | | | | | | | |
|---|---|---|---|---|---|---|---|---|---|---|---|---|---|
| | | n1 | n2 | Clusters v Edge intensity | | | Clusters v Visual Models | | | Edge intensity v Visual Models | | |
| | | | | Chi-sqr. | P | Sig* | Chi-sqr. | P | Sig* | Chi-sqr. | P | Sig* |
| Clades | | | | | | | | | | | | |
| A | B | 112 | 42 | 6.73 | 0.0345 | No | 9.8172 | 0.0074 | Yes | 9.7959 | 0.0075 | Yes |
| A | C | 112 | 175 | 30.99 | 0.0000 | Yes | 11.7701 | 0.0028 | Yes | 57.3548 | 0.0000 | Yes |
| A | D | 112 | 35 | 4.91 | 0.0857 | No | 5.0598 | 0.0797 | No | 1.1909 | 0.5513 | No |
| B | C | 42 | 175 | 43.23 | 0.0000 | Yes | 6.224 | 0.0445 | No | 49.8987 | 0.0000 | Yes |
| B | D | 42 | 35 | 5.370a | 0.0066 | Yes | 4.1256a | 0.0200 | No | 3.5028a | 0.0352 | No |
| C | D | 175 | 35 | 13.45 | 0.0012 | Yes | 2.9587 | 0.2278 | No | 18.8744 | 0.0000 | Yes |
| Locality | | | | | | | | | | | | |
| 1 | 2 | 52 | 52 | 5.33 | 0.07 | No | 5.4926 | 0.0642 | No | 3.57 | 0.17 | No |
| 1 | 3 | 52 | 52 | 4.6758 | 0.0965 | No | 25.7392 | 0.0000 | Yes | 17.075 | 0.0002 | Yes |
| 1 | 4 | 52 | 52 | 15.56 | 0.00 | Yes | 0.0259 | 0.9871 | No | 15.2006 | 0.0005 | Yes |
| 1 | 5 | 52 | 52 | 49.78 | 0.00 | Yes | 95.8278 | 0.0000 | Yes | 65.9819 | 0.0000 | Yes |
| 1 | 6 | 52 | 52 | 186.65 | 0.00 | Yes | 209.0618 | 0.0000 | Yes | 54.8152 | 0.0000 | Yes |
| 1 | 7 | 52 | 52 | 40.59 | 0.00 | Yes | 86.8951 | 0.0000 | Yes | 10.1971 | 0.0061 | No |
| 2 | 3 | 52 | 52 | 14.45 | 0.00 | Yes | 38.6709 | 0.0000 | Yes | 37.7307 | 0.0000 | Yes |
| 2 | 4 | 52 | 52 | 21.68 | 0.00 | Yes | 5.4267 | 0.0663 | No | 18.1406 | 0.0001 | Yes |
| 2 | 5 | 52 | 52 | 45.96 | 0.00 | Yes | 89.3429 | 0.0000 | Yes | 42.7745 | 0.0000 | Yes |
| 2 | 6 | 52 | 52 | 119.22 | 0.00 | Yes | 127.4365 | 0.0000 | Yes | 34.8508 | 0.0000 | Yes |
| 2 | 7 | 52 | 52 | 16.35 | 0.00 | Yes | 52.0487 | 0.0000 | Yes | 8.0936 | 0.0175 | No |
| 3 | 4 | 52 | 52 | 35.05 | 0.00 | Yes | 22.8218 | 0.0000 | Yes | 38.6415 | 0.0000 | Yes |
| 3 | 5 | 52 | 52 | 115.20 | 0.00 | Yes | 186.6832 | 0.0000 | Yes | 157.5528 | 0.0000 | Yes |
| 3 | 6 | 52 | 52 | 246.67 | 0.00 | Yes | 207.5054 | 0.0000 | Yes | 134.2205 | 0.0000 | Yes |
| 3 | 7 | 52 | 52 | 70.08 | 0.00 | Yes | 53.2656 | 0.0000 | Yes | 43.1209 | 0.0000 | Yes |
| 4 | 5 | 52 | 52 | 1.89 | 0.39 | No | 85.0204 | 0.0000 | Yes | 49.8415 | 0.0000 | Yes |
| 4 | 6 | 52 | 52 | 165.28 | 0.00 | Yes | 167.6656 | 0.0000 | Yes | 28.011 | 0.0000 | Yes |
| 4 | 7 | 52 | 52 | 48.38 | 0.00 | Yes | 60.6155 | 0.0000 | Yes | 3.3943 | 0.1832 | No |
| 5 | 6 | 52 | 52 | 144.03 | 0.00 | Yes | 341.0327 | 0.0000 | Yes | 6.6121 | 0.0367 | No |
| 5 | 7 | 52 | 52 | 71.64 | 0.00 | Yes | 296.8524 | 0.0000 | Yes | 53.3422 | 0.0000 | Yes |
| 6 | 7 | 52 | 52 | 58.20 | 0.00 | Yes | 32.69 | 0.0000 | Yes | 40.4267 | 0.0000 | Yes |
| Habitat | | | | | | | | | | | | |
| Coast | Other | 156 | 208 | 26.65 | 0.00 | Yes | 136.285 | 0.0000 | Yes | 30.4938 | 0.0000 | Yes |

a F-test with 2 and 74 degrees of freedom

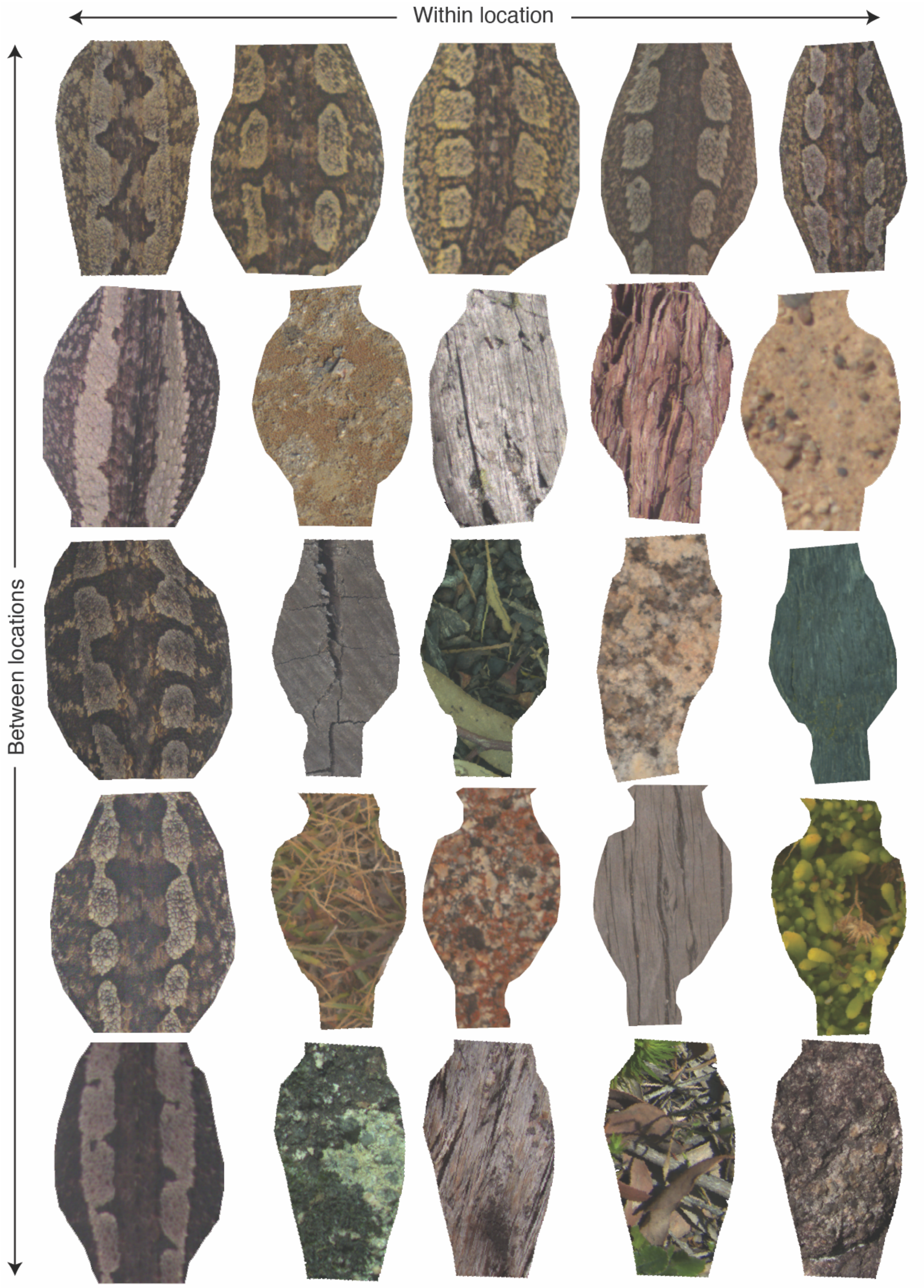


**Figure S1:** Illustration of pattern variation for both lizards and backgrounds. Images within a given row are from the same location, while different locations are represented in each row (top to bottom: Murramarang NP, Werrikimbe NP, You Yangs RP, Croajingalong NP, Gibraltar NP). The top row illustrates differences within location, while subsequent rows showcase different backgrounds. The shapes of the background images are indicative of the ROI of the lizard occupying the microhabitat in which the background was sampled.

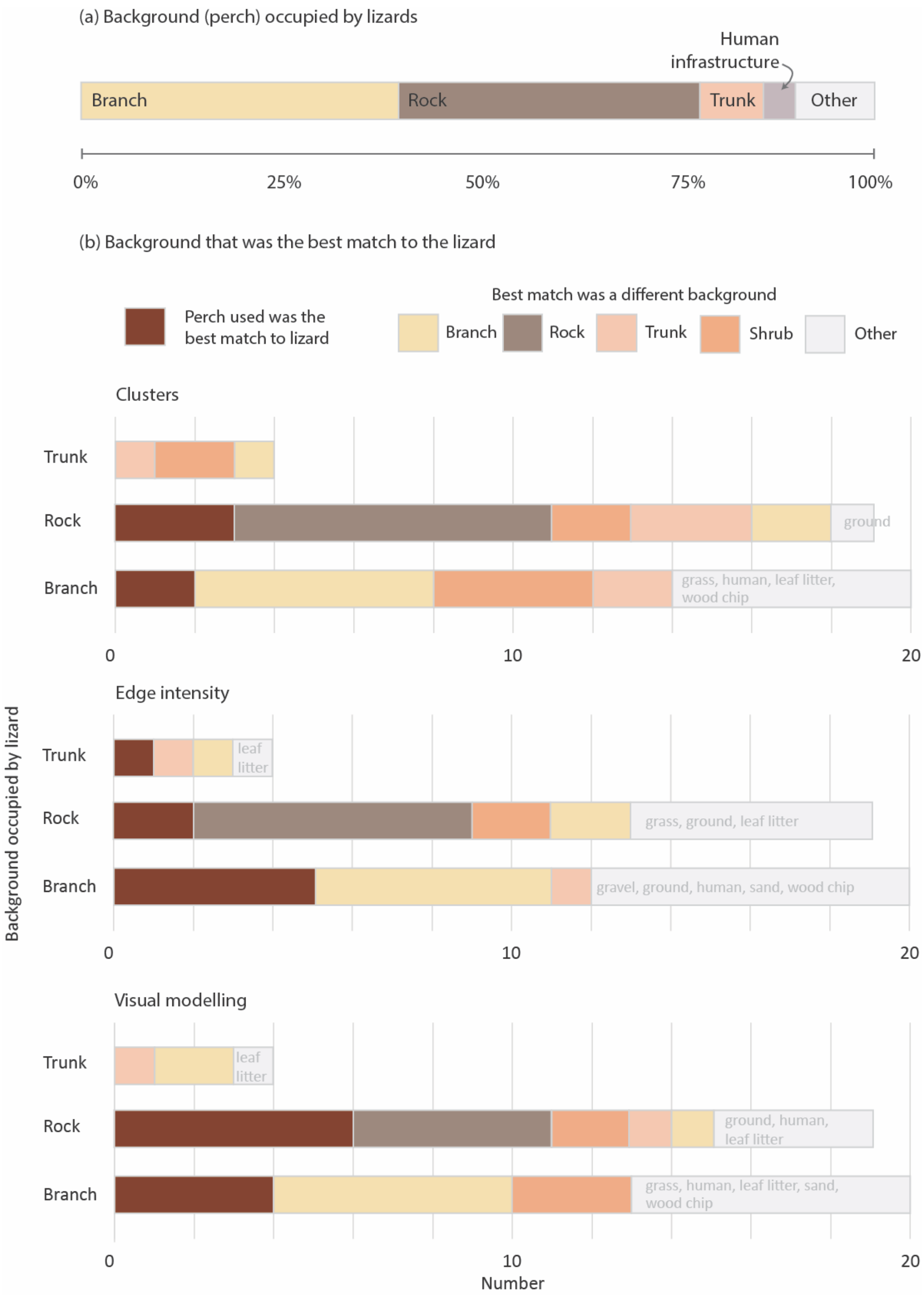


**Figure S2**: (a) Background (perch) occupied by lizards at the time of capture. Tree branches and rocks were the dominant background, along with perching on tree trunks. Human infrastructure included concrete and asphalt pathways, while other backgrounds included grass, gravel, ground, leaf litter, sand, shrub, wood chips. (b) The background in the vicinity of each lizard that had the best match to lizard patterns is identified for the three main backgrounds used by lizards: tree branches, rocks and tree trunks. Separate panels are provided for the different metrics used: clusters (top), edge intensity (middle) and visual modelling (bottom). In each case, the best matching background is identified as either the one occupied by the lizard or an alternative as indicated in the legend above the top panel.

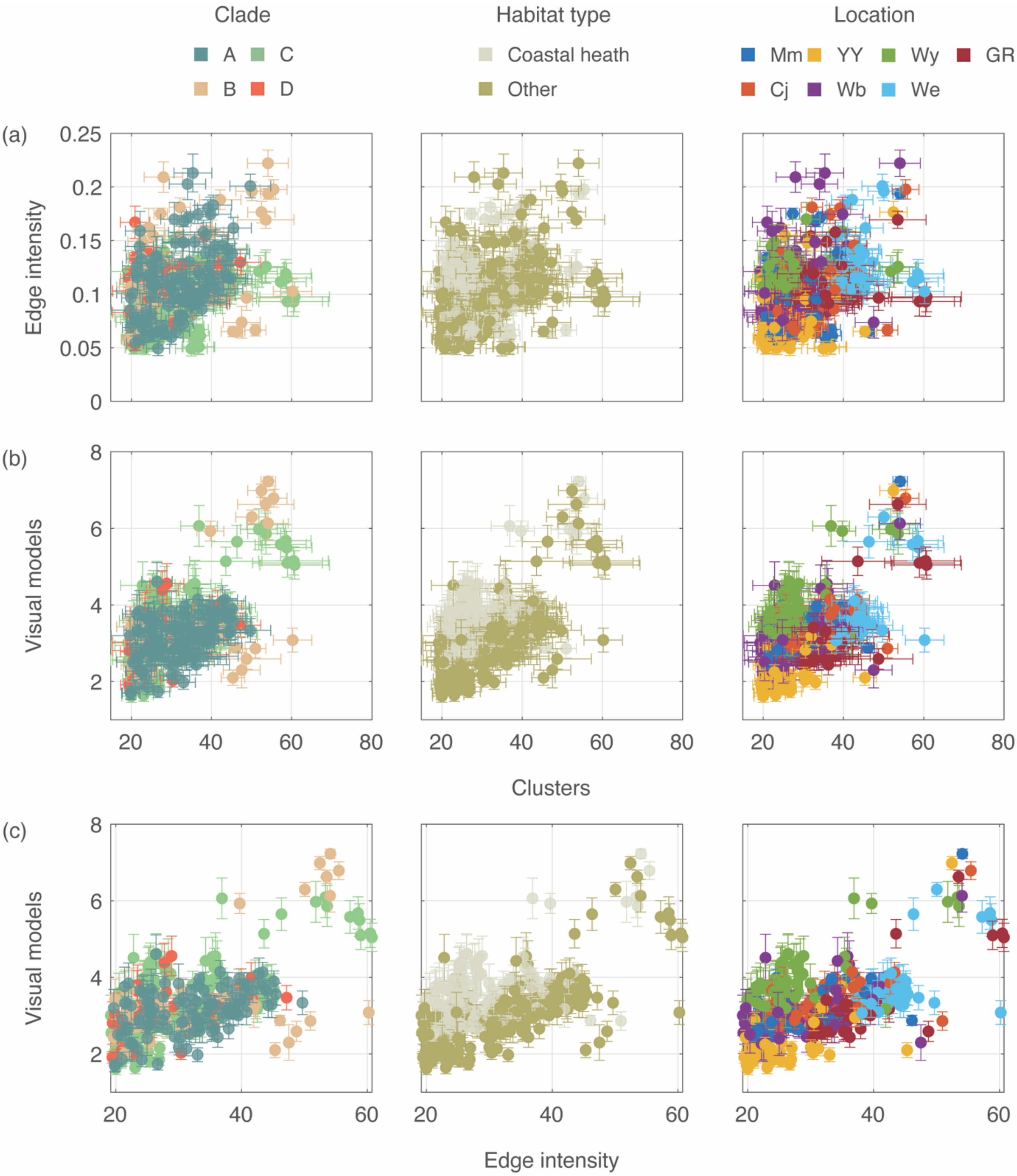


**Figure S3:** Multiple two-dimensional plots of the Euclidean distance between each lizard and backgrounds from all locations in the study as shown in Figure 4.6. Values are averaged within a given location for each lizard, while error bars represent standard error. The data are the same within a given row, but colour coded according to clade to which the lizard belongs (left column), as well as the habitat type and location of the background (middle and right columns respectively). Data is restricted to clusters and edge intensity (a), clusters and visual models (b) and edge intensity and visual models (c). Location abbreviations: Murramarang (Mm), Croajingalong (Cj), You Yangs (YY), Warrumbungles (Wm), Wyrrabalong (Wy), Werrikimbe (We) and Gibraltar Ranges (GR).

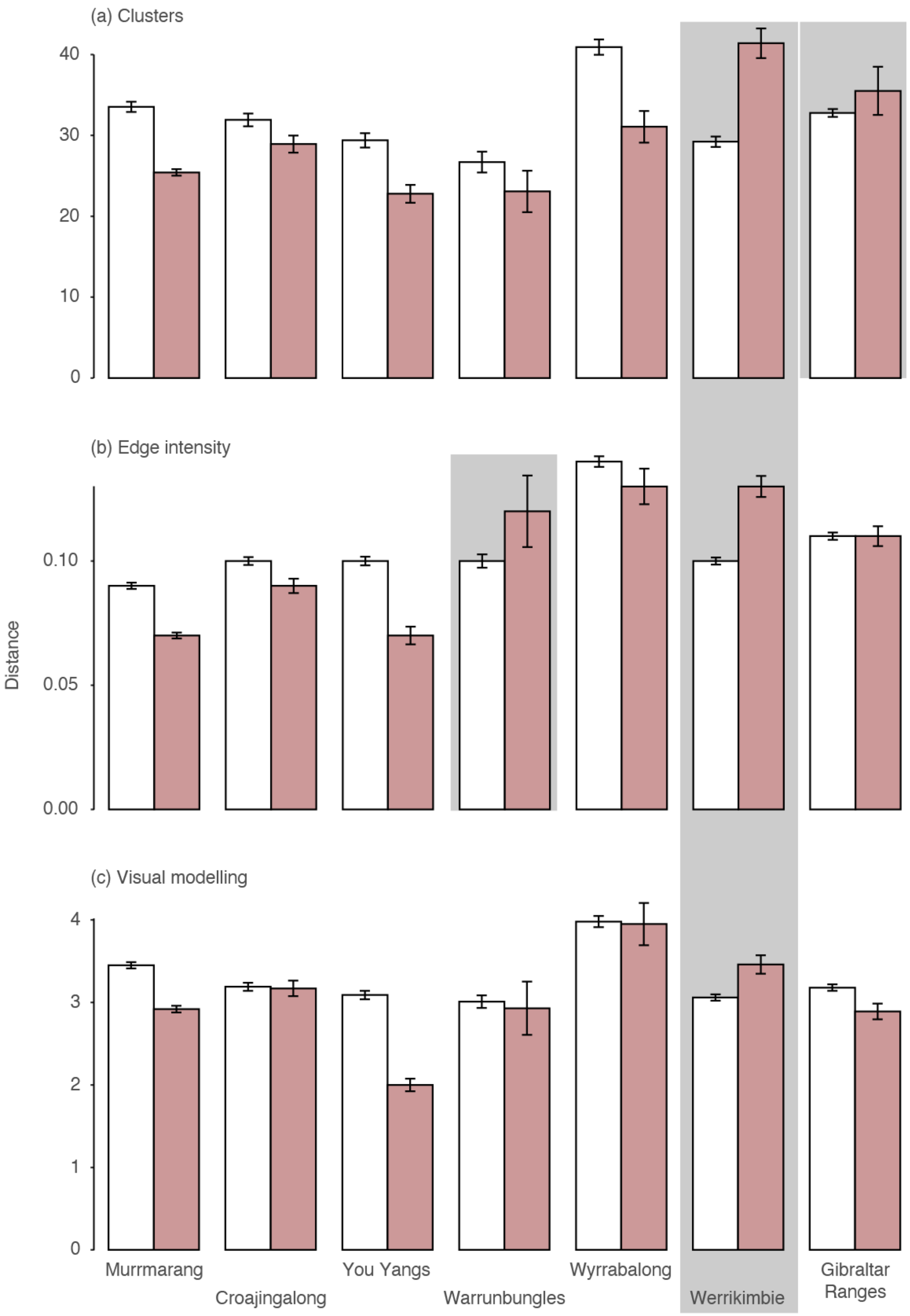


**Figure S4**: Mean (± SE) distance measures between lizard appearance and background summarised by location of the lizard and for backgrounds within (coloured) and outside (white) the location. Separate plots are shown for (a) cluster, (b) edge intensity and (c) visual modelling data. Gray background on plots denotes locations where distance within the location is greater than outside.
